# The Role of Tumor-associated Macrophages in Colorectal Peritoneal Metastasis in Mice

**DOI:** 10.14789/ejmj.JMJ24-0049-OA

**Published:** 2025-06-06

**Authors:** MEGUMI KAWAGUCHI, MASAYA KAWAI, SHINYA MUNAKATA, HARUNA ISHIHARA, YUKI TSUCHIYA, KUMPEI HONJO, KIICHI SUGIMOTO, MAKOTO TAKAHASHI, KAZUHIRO SAKAMOTO

**Affiliations:** 1 Department of Coloproctological Surgery, Juntendo University Faculty of Medicine, Tokyo, Japan; 1 Department of Coloproctological Surgery, Juntendo University Faculty of Medicine, Tokyo, Japan

**Keywords:** colorectal cancer, macrophage, peritoneal metastasis, tumor-associated macrophage, IL-10

## Abstract

**Introduction:**

In the tumor microenvironment, macrophages function as M1 macrophages, which cause cytotoxicity to tumor cells in the early stages, and M2 macrophages, which contribute to the proliferation of cancer cells in the late stages. This study aimed to examine the mechanism of action of macrophages in ascites using a peritoneal dissemination mouse model of colorectal cancer.

**Materials and Methods:**

Mouse models of peritoneal dissemination were created by injecting murine colorectal cancer cells into the abdominal cavity of non-obese diabetic/severely combined immunodeficient mice. Surface markers for M1 (CD38, CD68) and M2 (CD83, CD206) were used for macrophage differentiation via flow cytometry. Quantitative PCR and microarray gene expression analyses were performed on macrophages isolated from ascites. Additionally, a macrophage inhibitor (clodronate) and IL-10 inhibitor (AS101) were injected intra-abdominally, and the weight of peritoneally disseminated nodules was compared.

**Results:**

M1 macrophage counts showed no change over time, while M2 counts and the expression of arginase 1 and IL-10 increased. Clodronate administration significantly reduced the weight of peritoneally disseminated nodules. and AS101 administration reduced tumor size.

**Conclusion:**

Our findings indicate that cytokine and gene expression analyses of TAMs should be further explored to improve targeted anticancer therapy

## Introduction

Colorectal cancer (CRC) is the third most common malignancy and the second most common cause of cancer-related deaths globally^1)^. The peritoneum is one of the most frequent sites of metastasis in CRC, in addition to the liver and lung. Peritoneal metastases are associated with poor progression-free and overall survival. Macrophages are a highly plastic cell type that adapts to the stromal environment of malignant tumors. The dominant tumor-associated macrophage (TAM) phenotype is anti-inflammatory and immunoregulatory and therefore tumor- promoting (also termed alternatively activated or M2 macrophages), as opposed to pro-inflammatory and tumoricidal (classically activated or M1 macrophages). M1 and M2 macrophages are expected to express a common set of proteins involved in basic macrophage functions, such as macrophage-specific gene transcription regulation, protein synthesis, and phagocytosis^2-3)^.

The surface markers used to distinguish murine macrophages are CD38 and CD68 (M1) and CD83 and CD206 (M2)^4-7)^. M1 macrophages mostly produce pro-inflammatory molecules such as tumor necrosis factor-α (TNF-α), interleukin (IL)-6, IL-12, IL-23, and inducible nitric oxide synthase (iNOS). M2 macrophages secrete Th2-type cytokines, such as IL-4, IL-10, IL-13, and transforming growth factor (TGF-β), and show a pro-tumorigenic phenotype.

Recent studies have shown that the bisphosphonate macrophage inhibitor zoledronic acid significantly reduces the complications of bone metastasis^8-9)^ and that colony-stimulating factor (CSF)-1 inhibition depletes macrophages and reduces tumor volume in vivo and in vitro^10-11)^. The chemokine C- C chemokine receptor type 2 (CCL2) is responsible for the recruitment of C-C motif chemokine ligand 2 (CCR2)^+^ inflammatory monocytes from the bone marrow to the peripheral blood where they ultimately migrate to pancreatic cancer and become immunosuppressive TAMs. CCR2 blockade results in reduced TAM infiltration and promotes an endogenous antitumor immune response^12)^. As shown in these reports, the formation of a targeted TAM deletion state may regulate TAM-related cancers, and TAMs should be explored as a new anticancer therapeutic target in future clinical trials.

In this study, we investigated the mechanism of macrophages in mouse models of peritoneal dissemination and elucidated TAM-related factors.

## Materials and Methods

### Animal study design

Non-obese diabetic severe combined immunodeficient (NOD SCID) mice (NOD.CB17-Prkdc^scid^/J) were obtained from Charles River Laboratories. Under anesthesia, 1.0 × 106 CMT93 cells were injected into the peritoneal cavity of NOD SCID mice. On day 33, mice were dissected to examine the presence of peritoneal nodules.

To investigate the possible roles of macrophage subsets in CRC metastasis, we determined the dominant TAM type distributed in the peritoneal cavity using M1/M2 surface markers at 0, 7, and 33 days after the induction of peritoneal dissemination. Subsequently, M1/M2-related mRNA expression was evaluated in F4/80^+^ macrophages, targeting the M1 markers TNF-α and iNOS and M2 markers arginase 1 (ARG1) and IL-10. Three mice were dissected at each time point.

Gene expression was compared between normal macrophages and peritoneal TAMs. mRNA was isolated from peritoneal F4/80^+^ cells followed by gene-level differential expression analysis using the GeneChip™ 3' IVT Pico Kit (Applied Biosystems, Waltham, MA, USA) at 0 and 33 days following induction of peritoneal dissemination. Samples from three mice were obtained at each time point.

For the induction of macrophage depletion, 25 μL of liposome-encapsulated clodronate, Clophosome- N (F70101C-N, FormuMax Scientific, Sunnyvale, CA, USA), was injected intraperitoneally on days 1, 11, and 21 (control group: 3 mice, clodronate group: 5 mice). An IL-10 inhibitor AS101 (Tocris Bioscience, Bristol, UK) was injected (20 mg/mouse) on days 1-35 (control group: 4 mice, AS101 group: 3 mice). Subsequently, a weighted comparison between the two groups was conducted for each animal.

### Histology

Peritoneal tumors were fixed in 10% buffered formalin, embedded in parafﬁn, cut into 5-μm sections, and stained with hematoxylin and eosin.

### Immunohistochemistry

F4/80 is a cell surface protein and a well-known marker of macrophage populations in mice that is used for macrophage differentiation.

Peritoneal tumors were stained with rabbit anti-IL-10 receptor subunit alpha (IL-10RA) polyclonal antibody (MyBioSource, San Diego, CA, USA) and rabbit anti-IL-10 polyclonal antibody (Bioss Antibodies, Woburn, MA, USA).

### Flow cytometry

Cells in the peritoneal cavity responsible for adhesion were identified using fluorescence-activated cell sorting. Cells were collected from 3 mice per group after peritoneal lavage and stained with the following antibodies: CD14-PE, CD45-PerCP (both Tonbo Biosciences, San Diego, CA, USA), F4/80 (Bio-Rad, Hercules, CA, USA), CD38-FITC, CD68-APC-Cy7, CD206-PE-Cy7, and CD83-APC (all from BioLegend, San Diego, CA, USA). The mean rates of CD38^+^CD68^+^ and CD83^+^CD206^+^ cells among F4/80^+^ cells were compared over the experimental period.

### Transcription polymerase chain reaction

For quantitative polymerase chain reaction (qPCR), macrophages were sorted using ARIA III (BD Biosciences Franklin Lakes, NJ, USA). RNA was then isolated from macrophages using NucleoSpin RNA Plus (Takara Bio, Otsu, Japan) and amplified using RNA-direct™ SYBR^®^ Green Real-time PCR Master Mix (TOYOBO, Osaka, Japan), which directly amplifies RNA without generating cyclic deoxyribonucleic acid before reaction, on a 7500 Fast Real-Time PCR system (Thermo Fisher Scientific, Waltham, MA, USA) according to the manufacturer's instructions. Relative mRNA expression was calculated using the 2^−ΔΔCt^ method. Each sample was analyzed in triplicate. The following forward and reverse primer pairs were used (forward and reverse): β-actin (*Actb*) 5′-AGAGCTACGAGCTGCCTGAC-3′ and 5′-AGCACTGTGT TGGCGTACAG-3′, IL-10 5′-TCTGGTGAAGGAGGATCGCTA-3′ and 5′-TGGCAACCCAGGTAACCCTA-3′, iNOS (*Inos*) 5′-CCTTGTTCAGCTACGCCTTC-3′ and 5′-AAGGCCAAACACAGCATACC-3′, CCR2 (*Ccr2*) 5′-GGTCATGATCCCTATGTG G-3′ and 5′-CTGGGCACCTGATTTAAAGG-3′, *IL- 10* 5′-ATTTGAATTCCCTGGGTGAGAAG-3′ and 5′-CACAGGGGAGAAATCGATGACA-3′, TNF-α (*Tnf*) 5'-GCCGATTTGCTATCTCATAC-3′ and 5′-GGTATATGGGCTCATACCAG-3′, ARG1 (*Arg-1*) 5′-AACACGGCAGTGGCTTTAACC-3′ and 5′- GGTTTTCATGTGGCGCATTC-3′, transforming growth factor beta 1 (*Tgf-b1*) 5′-TGACGTCACTGGAGTTGTACGG-3′ and 5′-GGTTCATGTCATGGATGGTGC-3′, and *Actb* 5′-GGCTGTATTCCCC TCCATCG-3′ and 5′-CCAGTTGGTAACAATGCC ATGT-3′.

### Microarray data analysis

RNA was used to perform a microarray analysis following the kit protocol (GeneChip™ 3′ IVT Pico Kit). The Transcriptome Analysis Console (TAC) software (v.4.0) tool (Applied Biosystems) was used for data analysis.

### Statistical analysis

JMP version 14 software (SAS Institute, Cary, NC, USA) was used for statistical analyses. Values are represented as mean ± SEM, and significance levels are indicated as *P < 0.05, **P < 0.01, ***P < 0.001 as calculated using the Mann-Whitney U test.

## Results

### M2 macrophages migrate into the peritoneal cavity during cancer progression

**Figure 1:**
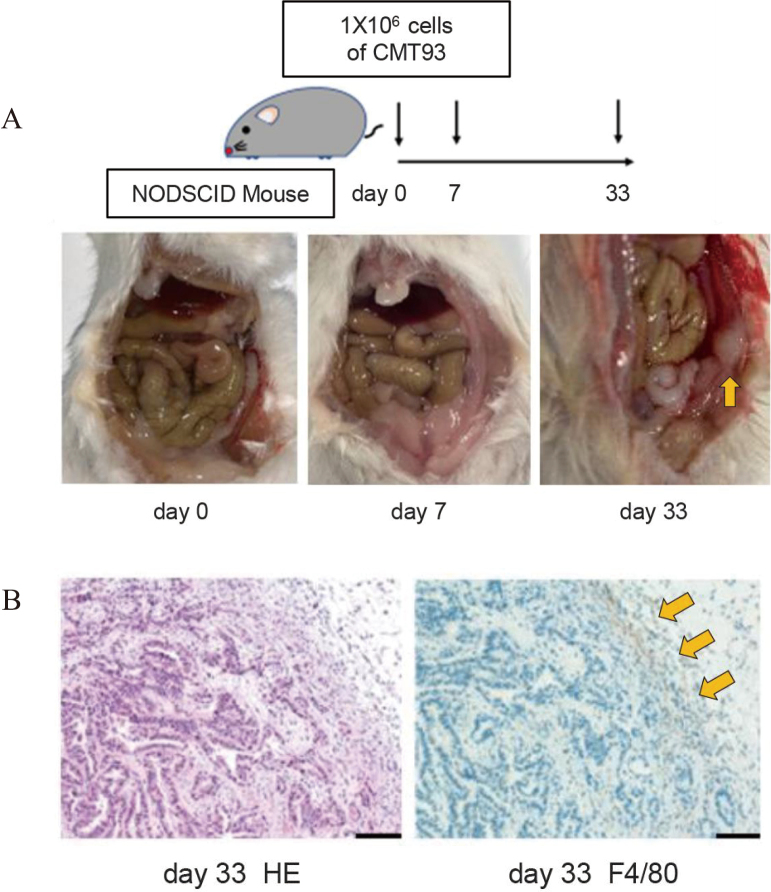
(A) Protocol for CMT93 injection into NOD SCID mice. Representative macroscopic images of peritoneal metastasis. Arrow: disseminated peritoneal nodule. (B) Representative hematoxylin and eosin and F4/80 immunofluorescent staining images of tumor samples retrieved at 33 days. Scale: 100 μm. Arrows: F4/80 staining (macrophage marker)

**Figure 2:**
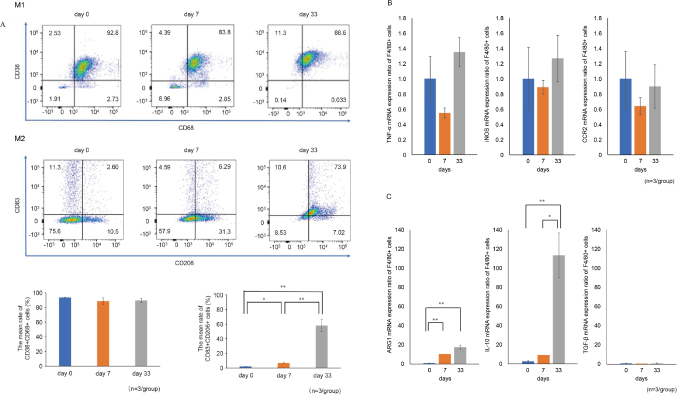
(A) One representative flow cytometry analysis is shown. M2 but not M1 cell counts showed a significant change over the experimental period (day 0 to day 33). (B) Expression of M1 macrophage markers TNF-α, iNOS, and CCR2 did not increase with cancer progression. (C) Expression of M2 macrophage markers (ARG1 and IL-10, but not TGF-β) was increased with cancer progression.

Peritoneal nodules were detected in mice dissected on day 33 (Figure 1A) as well as a massive accumulation of F4/80^+^ cells in the cancer tissue (Figure 1B). Additionally, F4/80^+^ macrophages were recruited to the peritoneal fluid at 0, 7, and 33 days after the induction of peritoneal dissemination. A significant difference in M2 cell count but not M1 cell count was found during tumor growth from day 0 to day 33 (Figure 2A). TNF-α, iNOS, and CCR2 expression levels were not related to M1 cell counts. However, M2 ARG1 and IL-10 but not TGF-β expression levels increased with cancer progression (Figure 2B, C).

### Macrophage depletion prevents peritoneal metastatic progression

**Figure 3:**
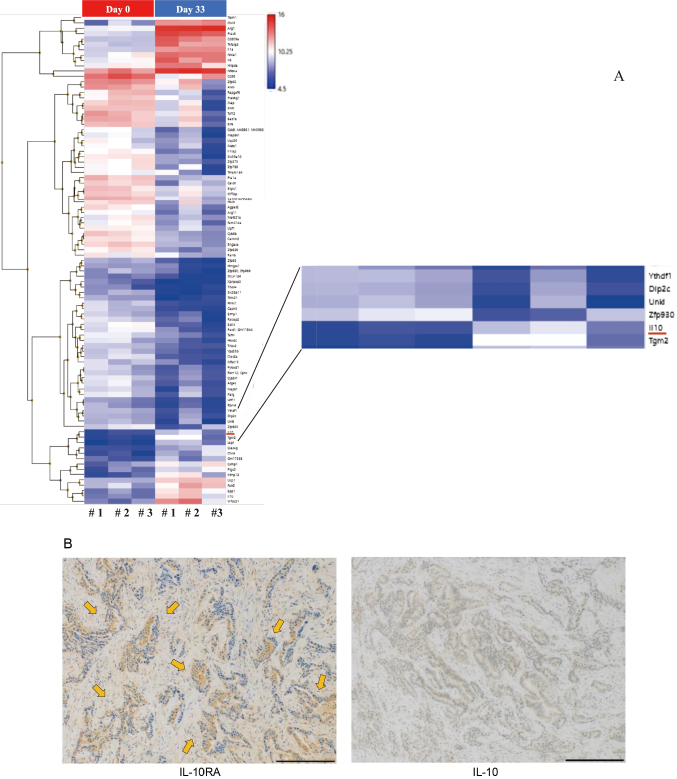
(A) Heatmap of differentially expressed genes in peritoneal F4/80+ macrophages from mice injected with CMT93. IL-10 expression increased over five times in ascites of mouse models. Arrow: IL-10 (B) Representative IL-10RA and IL-10-stained sections of peritoneal metastasis tissue are shown. Scale: 200 μm.

**Figure 4:**
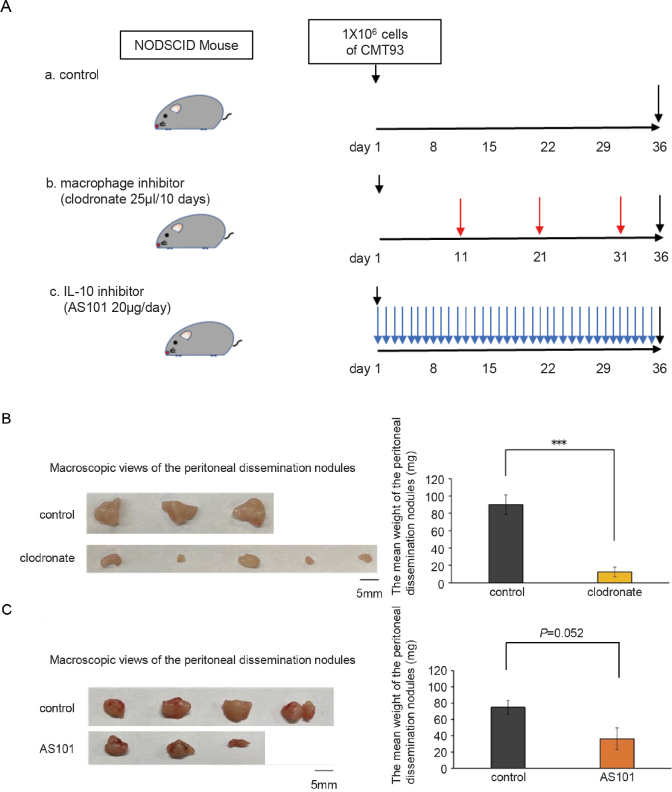
(A) Protocol for macrophage inhibitor (clodronate) and an IL-10 inhibitor (AS101) injection into NOD SCID mice. (B) Macroscopic views and the mean weight of the peritoneal dissemination nodules from animals injected with clodronate and negative controls. The administration of clodronate significantly reduced the size of peritoneally disseminated nodules (P < 0.001). (C) Macroscopic views and the mean weight of the peritoneally disseminated nodules from animals injected with AS101 and negative controls.

A heat map of the calibrated samples is shown in Figure 3A. The expression of IL-10 was visibly increased in the ascites of mice. Therefore, we focused on IL-10 expression. IL-10 and IL-10RA immunostaining indicated that IL-10 was not expressed by tumors; however, IL-10RA expression was highly localized in tumor cells (Figure 3B). Furthermore, clodronate administration significantly reduced the weight of peritoneal nodules (P < 0.001) (Figure 4A, B). Additionally, AS101 inhibited cancer progression. However, this difference was not statistically significant (P = 0.052) (Figure 4C).

## Discussion

Although the survival of patients with CRC of all stages has improved, peritoneal dissemination occurs in 8-15% of patients, with a poorer prognosis than that associated with other sites of metastases^13)^. Therefore, an accurate assessment of peritoneal dissemination is vital for clinical decision-making and CRC prognosis improvement. Moreover, the standard treatment for peritoneal dissemination is based only on systemic chemotherapy, and surgery is considered exclusively for palliative purposes. Other therapies for preventing peritoneal dissemination in patients with CRC include cytoreductive surgery (CRS) and hyperthermic intraperitoneal chemotherapy. However, the PRODIGE 7 randomized controlled trial did not demonstrate an overall survival benefit associated with CRS plus hyperthermic intraperitoneal chemotherapy compared with CRS alone^14)^. With regard to systemic chemotherapy, validated biomarkers such as v-Ki-ras2 Kirsten rat sarcoma viral oncogene homolog (KRAS), v-Raf murine sarcoma viral oncogene homolog B1 (BRAF), and microsatellite instability (MSI) cover the extensive heterogeneity of CRC and are suitable for guiding personalized treatment. To improve the care of patients with CRC with peritoneal dissemination, better molecular characterization is required^15)^.

By demonstrating increased F4/80^+^ macrophage levels in a peritoneal dissemination mice model, this study may contribute to the molecular and genetic understanding of CRC and treatment focused on cell analysis. Yamaguchi et al.^16)^ isolated a large quantity of TAMs expressing CD163 and CD204 from patients with late-stage gastric cancer with peritoneal dissemination. Eum et al.^17)^ showed that in gastric cancer most TAMs express proinflammatory cytokine/chemokine genes, such as IL1B, CCL2, CCL3, and CCL20. In that study, the most abundant cytokine-receptor pair interaction between TAMs and tumor cells was predicted for IL1B and its decoy receptor IL1R2, suggesting inhibition of IL1B-mediated proinflammatory signaling by tumor cells. Additionally, the interaction between IL10 and IL10RA within TAM populations limited inflammatory immune responses. Therefore, we also focused on IL-10 and IL-10RA in a peritoneal dissemination mouse model because IL-10R is highly expressed in peritoneal nodules. In our study, the administration of a macrophage inhibitor reduced the size of peritoneally disseminated nodules, and the administration of an IL-10 inhibitor reduced the number of tumors.

Oshima et al.^18)^ showed that a macrophage inhibitor eliminates TAMs in mouse models of gastric cancer. Additionally, Bader et al.^19)^ demonstrated that macrophage inhibitors deplete TAMs and decrease tumorigenesis in mouse models of colon cancer. Further research on macrophage inhibitor- mediated TAM depletion mechanisms may provide insights relevant to novel treatments.

Although IL-10 has anti-inflammatory effects, it may also stimulate tumor immunity at high concentrations^20)^. A pegylated human IL-10 that activates tumor immunity^21)^, pegilodecakin (PEG), in combination with folinic acid, fluorouracil, and oxaliplatin (FOLFOX) has been evaluated in a phase III trial (SEQUOIA study) as a second-line treatment for patients with metastatic pancreatic cancer, but its improved efficacy was not demonstrated^22)^. However, IL-10 has several actions and may have conflicting effects depending on dosage and the patient. Although these trials have failed to show efficacy, the treatment regimens utilized may be effective in patients with peritoneal dissemination or increased macrophages. Chen et al.^23)^ reported that IL 10- expression was upregulated in gastric tumor tissues and serum of patients with gastric cancer and that IL-10 was increased in the cell culture supernatant of central nervous system-associated macrophages (CAMs). Furthermore, treatment with CAM cell culture supernatants induced an increase in proliferation and migration, while suppressing MGC-803 and BGC-823 gastric cancer cell apoptosis. Factors promoting M1/M2 polarization have been reported, and further examining the effect of macrophage cell culture supernatants on tumors may be worthwhile. While this is a topic for future research, a more multifaceted approach for targeting macrophages and IL-2 should be developed to combat cancer cells more effectively than current methods.

In conclusion, this study showed that the M2 macrophage population increases in a mouse peritoneal dissemination model of CRC, and macrophage inhibitors effectively suppress peritoneal dissemination. IL-10 inhibitors also suppress cancer progression. Therefore, further cytokine and gene expression analyses of TAMs may aid in improving targeted anticancer therapy.

## Funding

This study was partially supported by a Grant-in-Aid for Scientific Research from the Japan Society for the Promotion of Science (Wakate Grant 19K1047840, SM).

## Availability of data and materials

The datasets used and analyzed in the current study are available from the corresponding author upon reasonable request.

## Author contributions

Data acquisition (MK, HI), drafting of the manuscript (SM), critical revision of the manuscript (YT, KH, MK, KS, MT, and KS).

## Ethics approval and consent to participate: H16-0157

Animal studies were approved by the Animal Review Board of Juntendo University.

## Patient consent for publication

Not applicable.

## Conflicts of interest statement

The authors declare that there are no conflicts of interest.

## Authors' information (optional)

Not applicable.
